# Impact of Environmental Food Intake on the Gut Microbiota of Endangered Père David’s Deer: Primary Evidence for Population Reintroduction

**DOI:** 10.3390/ani14050728

**Published:** 2024-02-27

**Authors:** Qiying Mo, Hongyu Yao, Hong Wu, Dapeng Zhao

**Affiliations:** 1 Tianjin Key Laboratory of Conservation and Utilization of Animal Diversity, College of Life Sciences, Tianjin Normal University, Tianjin 300387, China18202676033@163.com (H.Y.); 2 College of Life Sciences, Beijing Normal University, Beijing 100875, China

**Keywords:** *Elaphurus davidianus*, reintroduction, dietary composition, bacterial community, fecal microscopic analysis

## Abstract

**Simple Summary:**

Comprehensive individual health assessments are required following reintroduction to enhance ex situ species conservation. Diet directly affects the health of wildlife, which can be monitored by fecal microbe analysis. In this study, the association between the gut microbiota and the dietary components of a wild Père David’s deer (*Elaphurus davidianus*) population was evaluated. It was shown through 16S rRNA high-throughput sequencing that the gut microbiota was dominated by Firmicutes and Proteobacteria, with *Psychrobacillus* accounting for the largest proportion at the genus level. Microscope observations showed that the relative density of *Nymphoides peltata* in the fecal matter was significantly positively correlated with the abundance of Firmicutes. This study provides in-depth insight into the relationship between dietary composition and gut microbiota of wild *E. davidianus*, which offers guidance for the reintroduction of this endangered species to its historical habitat in China.

**Abstract:**

Reintroduction has been successful in re-establishing several endangered wild animals in their historical habitats, including Père David’s deer (*Elaphurus davidianus*). Continuous monitoring of reintroduced individuals is essential for improving the sustainability of ex situ conservation efforts. Despite an increased recognition of the significance of the gut microbiome for animal health, the correlation between diet and the gut microbiome in *E. davidianus* is unclear. In this study, 15 fresh fecal samples of *E. davidianus* were collected from Tianjin Qilihai Wetland and the association between dietary and gut microbiota composition was evaluated. Microscopic observations showed that *Nymphoides peltata* [relative density (RD = 0.3514), *Phragmites australis* (RD = 0.2662), *Setaria viridis* (RD = 0.1211), and *Typha orientalis* (RD = 0.1085) were the main dietary plants in the fecal samples. High-throughput 16S rRNA sequencing showed a predominance of the phyla Firmicutes and Proteobacteria and the genus *Psychrobacillus* (26.53%) in the gut microbiota. The RD of *N. peltata* was significantly positively correlated with the abundance of Firmicutes (*p* = 0.005) and the genus *UCG-005* (*p* = 0.024). This study indicates a close association between food digestion and nutrient intake, providing basic monitoring data for the full reintroduction and recovery of wild *E. davidianus*.

## 1. Introduction

Reintroduction, which involves the release of captive-bred individuals to the original habitat from which they were extirpated [1,2,3], has been widely used to recover populations of endangered wildlife in the field of conservation biology [1]. This strategy has been successful in enabling the reappearance and breeding of several endangered wildlife species in their historical habitats [4].

The Père David’s deer (*Elaphurus davidianus*) is one of the successful examples of wild population recovery through reintroduction [5,6]. The Père David’s deer is an endemic species of China from the early Pleistocene [7], which disappeared around 1900 and was classified as “extinct in the wild” on the International Union for Conservation of Nature Red List [8]. Père David’s deer from the United Kingdom were then released into their historical habitat as a reintroduction strategy in 1985–1986 [7], and the population has gradually become accustomed to the wild habitat in China, including Hubei, Hunan, and Tianjin [5]. Although the total population of Père David’s deer in China is now close to 10,000 [6], most individuals are still in captivity and there is still a long way to go to facilitate their reproduction in the natural environment to help the population revert to the original state [9]. Various factors have been reported to have a significant influence on the success of reintroduction, including the quality of the habitat [10], the source of reintroduced individuals [11], pre-reintroduction behavioral training [12], and appropriate scientific monitoring during reintroduction [2,3,13]. In particular, dietary monitoring enables researchers to gain insights into the impact of new environments on the viability and health status of wildlife, given the link between dietary habits and the gut microbiota composition, thereby providing a valuable reference for subsequent conservation interventions to facilitate their acclimatization to the wild [13,14,15].

There are various methods of monitoring dietary habits, including direct observations [16], rumen/gut contents analysis [17], stable carbon and nitrogen isotope analysis [18], and fecal microscopic analysis [19]. The latter method is particularly suitable for monitoring the diet of individuals as a non-invasive sampling technique that only requires collecting the excreta or feces to identify the composition and percentage of food ingested by animals in the wild, without disturbing the animals’ daily activities [20].

However, there have been few studies on the dietary habits of Père David’s deer to date. Wang et al. applied fecal microscopic and stable isotope analyses to investigate the staple food plants of Père David’s deer in summer and winter from three survival modes in Jiangsu Dafeng Milu National Nature Reserve [19]. Subsequently, Wang et al. used stable isotope technology to analyze the diet composition of Père David’s deer in Hubei Shishou Milu National Nature Reserve [21]. The results showed that the diet was dominated by C_3_ grasses in autumn and winter, identified from fur and fecal samples, whereas the summer diet was dominated by C_3_ forbs, identified from muscle and liver samples [21].

Conservation biology research applies a wide range of morphological [22], biochemical [23], and physiological metrics [24] to assess the health status of individual animals. Unlike in captive individuals, health status evaluation in wild animals should be carried out without disturbing their normal activities. Thus, the analysis of fecal samples based on dietary fragments and gut microbiota could be used to evaluate the health status of individuals, including parasite epidemiological investigations [25] and gut microbiota monitoring [26]. Gut microbiota play a crucial role in promoting food digestion and disease immunity [27]. Many factors, including age, sex, and living environment, can influence the diversity of intestinal bacteria, with a strong influence of diet [28]. Hence, gut microbiota monitoring based on non-invasive sampling techniques can be used to evaluate the adaptability of the host animal to its environment [19], offering a suitable strategy for the scientific monitoring of the health status of endangered species in the wild [29]. Although the gut microbiota of Père David’s deer have been studied, more work is needed to further explore the relationship between the gut microbiota and diet composition [26].

Establishing links between diet and the gut microbiome is important since the gut bacteria could modify the immune responses of wildlife to contribute to overall health. As diet composition is a principal determinant of gut microbial diversity, variation in the diet could lead to a change in microbiome diversity. However, the limited data of comparisons between dietary and microbiome diversity for wild animals have impeded efforts to explain the causes of observed gut microbiome variations. Therefore, in this study, we evaluated the association between the types of plants present in the fecal samples and the composition of the gut microbiomes of Père David’s deer living in Tianjin Qilihai Wetland, using microscopy and 16S rRNA high-throughput sequencing technology, respectively. This first exploration of the correlation between available foods and gut bacterial community composition could provide valuable basic data to facilitate the comprehensive reintroduction of this endangered species in the wild, while serving as a guide for further investigations into the factors contributing to the variation in gut microbiome composition.

## 2. Materials and Methods

### 2.1. Ethics Statement

The collection of fecal samples from Père David’s deer was permitted by Tianjin Qilihai Wetland without human disturbance to the animals. The non-invasive sampling strategy did not involve hunting or otherwise manipulating the experimental animals.

### 2.2. Sample Collection

A total of 15 fecal samples (samples SF01–SF15) were collected in late November 2021 from Tianjin Qilihai Wetland using a non-invasive sampling method [26]. The time node of the sample collection was after the traditional Chinese solar term the Beginning of Winter (7 November 2021). The fecal samples were collected immediately after observing the movement of Père David’s deer, as soon as they left the area. The collected fecal samples were sterilized in 5-mL tubes, temporarily stored in a refrigerated insulated tank, and then taken back to the laboratory for storage at −80 °C until analysis.

A total of 30 species of plants (Table S1) from 15 families and 28 genera were collected in Tianjin Qilihai Wetland for identification and to analyze the diet of Père David’s deer based on the composition of the fecal samples. Plant samples were placed in envelopes and then taken back to the laboratory for further taxonomic identification according to the Illustrated Book of Higher Plants of China [30].

### 2.3. Pretreatment of Residues in Fecal and Plant Samples

The proportions of plant species in the fecal samples of Père David’s deer were analyzed with fecal microscopy. All 15 fecal samples and different tissue samples from the 30 collected plant species were placed in a blast drying oven (BluePard, Shanghai, China) at 60 °C and dried to a constant weight. After drying, the samples were then ground with liquid nitrogen into a powder, and 1 g of the powder was placed in a sodium hypochlorite solution until the sample was completely immersed for 3–5 h. The sample was then washed with distilled water through a 200-mesh filter sieve until the cell morphology of the component plants became clearly visible in the dish [19]. Finally, the samples were stained with a 0.01% methylene blue solution for 15 min and washed with distilled water for microscopic observation.

### 2.4. Microscopic Observation and Calculation

Three microscopic slides were prepared for each sample, which were observed with a microscope (Leica, Wetzlar, Germany) under 100× magnification to record the morphology of all the plant cuticle fragments in each sample. Identification of the plant species in the fecal samples was based on the established local plant cell morphological atlas database [31]. After identifying recognizable plant fragments, the epidermal cuticle fragments appearing in one field of vision were recorded as a single plant.

Relative density (RD) was then used as the metric for quantifying the food composition, which was calculated according to Equation (1):

RD = (D*_i_*/ΣD) × 100% (1)

where D represents the average density and D*_i_* is the average density of dietary species *i* determined in each field of view, calculated from the frequency of each plant in 60 fields of view (F) according to Equation (2):

D*_i_* = −ln (1 − F/100). (2)

According to the RD value, the food of Père David’s deer could be divided into three categories: the main food type (RD ≥ 10%), a common food type (1% ≤ RD < 10%), and an occasional food type (RD < 1%).

### 2.5. 16S rRNA High-Throughput Sequencing Analysis

The total DNA of all fecal samples was extracted using the TIANamp fecal DNA kit (Tiangen, China). After extraction, a polymerase chain reaction (PCR) amplification of the 16S rRNA gene V3-V4 hypervariable region was conducted using the primers 338F (5′ACTCCTACGGGAGGCAGCA3′) and 806R (5′GGACTACHVGGGTWTCTAAT3′). PCR amplification was carried out in triplicate with 10 ng of sample DNA, 10 μL Taq PCR Master Mix (2×), 0.8 μL of each primer (5 μM), and certified water to reach the total reaction volume of 20 µL. The PCR conditions were as follows: 98 °C for 3 min (initial denaturation); 30 cycles of 95 °C for 30 s (denaturing); 55 °C for 30 s (annealing), and 72 °C for 45 s (extension); and, finally, 72 °C for 10 min. The DNA library was sequenced on the Illumina MiSeq platform by Majorbio Bio-pharm Technology Co., Ltd. (Shanghai, China).

The raw FASTQ data of all samples were demultiplexed and low-quality base pairs were removed from the paired-end reads. QIIme (version 1.9.1) was applied to process the raw data. Sequences with 97% similarity were clustered using UPARSE (version 11) for the identification of operational taxonomic units (OTUs).

### 2.6. Correlation between Diet and Gut Microbiota Composition

SPSS 26.0 software was applied for statistical analysis to explore the potential correlation between diet composition and dominant microbial phyla/genera identified from the gut, based on Spearman’s rank coefficient.

For the 15 individuals, each sample type of plant RD was fitted and Spearman’s rank correlation analysis was performed of genera with a relative abundance greater than 1% and the top five phyla to assess the link between diet and the major dominant phyla/genera (*p* > 0.05 = no significant correlation; *p* < 0.05 = a significant correlation).

The correlation between the plant RD and the major dominant microbiota was deemed significant and highly significant at *p* < 0.05 and *p* < 0.01, respectively. Correlation plots and fitting curves were constructed using Origin 2023b software.

## 3. Results

### 3.1. Characteristics of Plant Cuticle Fragments

A total of 45 slides of fecal samples were prepared for microscopic identification of dietary components according to the micromorphology of plant cuticle fragments, including the characteristics of the stomata, the arrangement and size of epidermal cells, and the appearance of other epidermal structures. A total of 900 photographs were taken from the 15 fecal samples of Père David’s deer at 100× magnification. Reference slides of the leaf epidermis and other vegetative material were prepared for all the plant species occurring within the foraging range of Père David’s deer.

**Figure 1:**
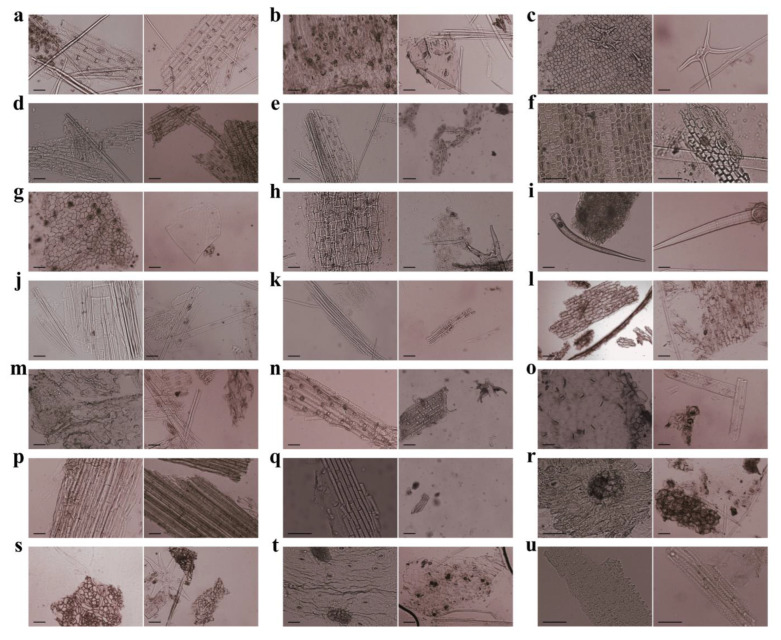
Microscopic photographs of original plant cells as reference (left) and residual plant cells from fecal samples (right). The plant species observed included (**a**) *Carex rigescens*, (**b**) *Medicago sativa*, (**c**) *Nymphoides peltate*, (**d**) *Phragmites australis*, (**e**) *Setaria viridis*, (**f**) *Typha orientalis*, (**g**) *Rumex patientia*, (**h**) *Myriophyllum spicatum*, (**i**) *Humulus scandens*, (**j**) *Suaeda glauca*, (**k**) *Bolboschoenus yagara*, (**l**) *Solanum nigrum*, (**m**) *Kochia scoparia*, (**n**) *Echinochloa crus-galli*, (**o**) *Chenopodium album*, (**p**) *Polygonum orientale*, (**q**) *Digitaria sanguinalis*, (**r**) *Metaplexis japonica*, (**s**) *Polygonum aviculare*, (**t**) *Artemisia carvifolia*, and (**u**) *Chloris virgata*. Bar: 50 µm.

Comparison of the collected plant species with the plant cell micromorphological atlas database resulted in the identification of a total of 19 species and 18 genera from 11 families in the fecal samples of Père David’s deer living in Qilihai Wetland (Figure 1). Among these plants, the common families identified were Poaceae, Gentianaceae, Typhaceae, Polygonaceae, and Chenopodiaceae, and the common genera were *Nymphoides*, *Phragmites*, *Setaria*, *Typha*, and *Rumex.*

### 3.2. Composition of Fecal Residues from Père David’s Deer

After the fecal samples were loosely broken up and bleached for the diet analysis, the fragments were separated and those with a sufficient cuticle to observe epidermal features were included in the final count. Among the 21 plant species collected from the study area, only two could not be identified in the fecal samples (representing 9.5% of all fragments analyzed).

**Figure 2:**
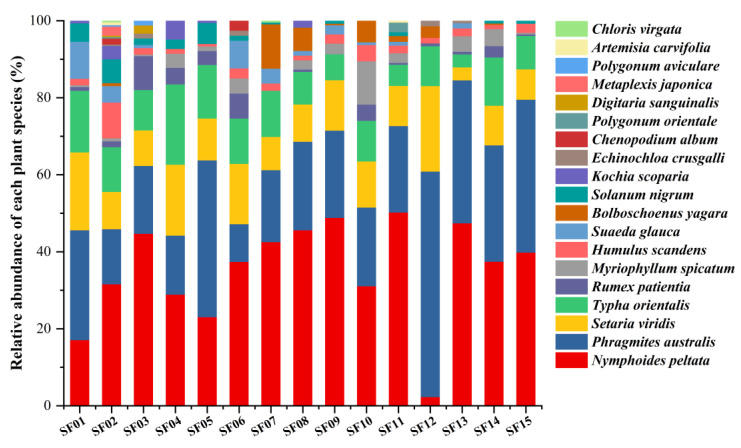
Relative density (RD) values of various identified plant species in the fecal samples of Père David’s deer (SF01–SF15).

Based on observations and histological analysis of the fecal samples, the main foods included *Nymphoides peltata*, *Phragmites australis*, *Setaria viridis,* and *Typha orientalis*, followed by *Rumex patientia*, *Myriophyllum spicatum*, *Humulus scandens*, and *Suaeda glauca*. According to the RD values of the identified plant species, there were four main foods pinpointed as those consumed by Père David’s deer: *N. peltata*, *P. australis*, *S. viridis*, and *T. orientalis*, which accounted for 84.72% of the total food (Figure 2, Table 1).

### 3.3. Gut Microbiota Diversity

High-throughput sequencing was performed on 16S rRNA of the 15 fecal samples collected in November, and a total of 3,830,746 sequences with an average length of 420 bp were obtained (Table S2). According to the classification based on 97% similarity, 2377 OTUs were obtained.

**Figure 3:**
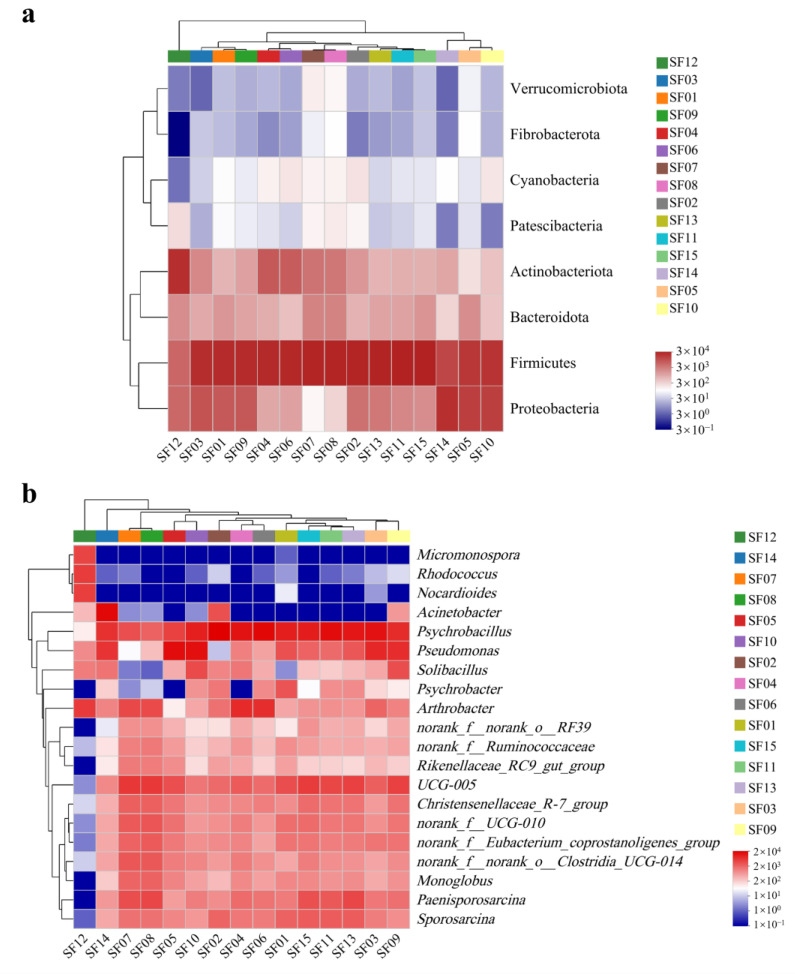
Composition of the gut microbiota from Père David’s deer (SF01–SF15) at the phylum (**a**) and genus (**b**) levels. The color intensity varies from blue to red, with red indicating more reads and blue indicating fewer reads.

The gut microbiota could be identified into 19 phyla, 48 classes, 88 orders, 145 families, and 235 genera. The dominant intestinal bacterial phylum of Père David’s deer from Tianjin Qilihai Wetland were Firmicutes (68.02%) and Proteobacteria (17.74%), followed by Actinobacteriota (10.09%) and Bacteroidota (3.47%). Fifteen bacterial genera accounted for more than 1% of the total, with the highest relative abundance represented by *Psychrobacillus* (26.53%), followed by *Pseudomonas* (11.33%), *UCG-005* (7.98%), *Arthrobacter* (6.22%), *Paenisporosarcina* (4.58%), *Acinetobacter* (3.71%), *Sporosarcina* (3.42%), *norank_f__UCG-010* (3.04%), *norank_f__Eubacterium_coprostanoligenes_group* (2.80%), *Christensenellaceae_R-7_group* (2.77%), *norank_f__norank_o__Clostridia_UCG-014* (2.41%), *Solibacillus* (2.29%), *Monoglobus* (1.91%), *Psychrobacter* (1.26%), and *norank_f__Ruminococcaceae* (1.17%) (Figure 3).

### 3.4. Potential Correlation between Food Composition and Gut Microbiota

**Figure 4:**
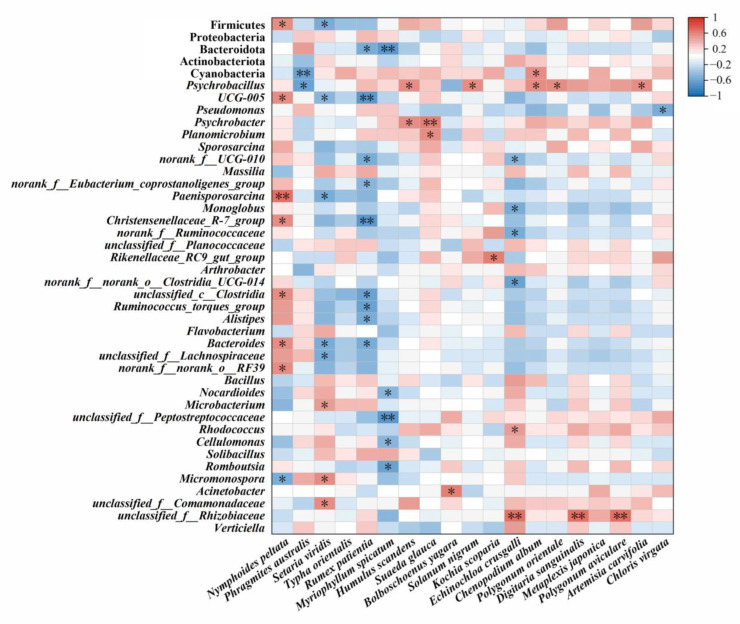
Potential correlation between edible plant relative density (RD) and abundance of gut microbiota based on Spearman’s rank correlation analysis. Red indicates a positive correlation and blue indicates a negative correlation. Stronger correlations are represented by the depth of color closer to the ends of the scale. * *p* < 0.05; ** *p* < 0.01.

**Figure 5:**
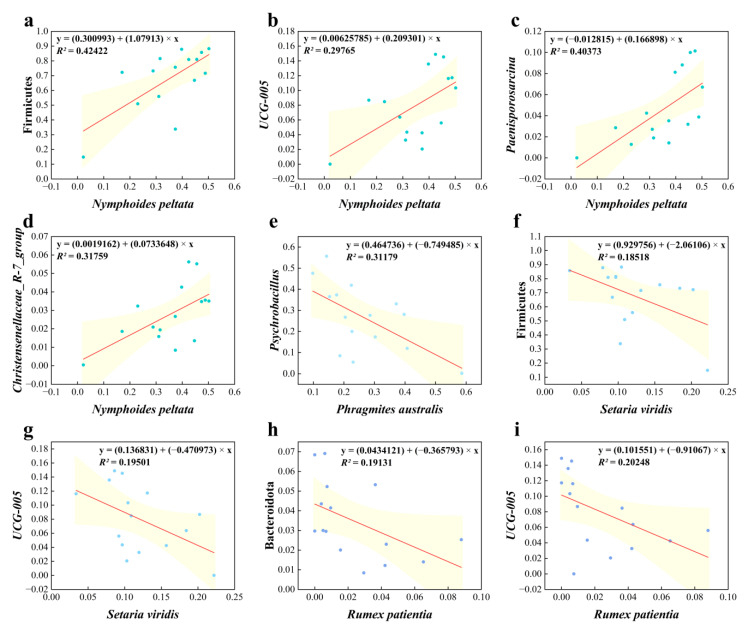
Potential correlation between edible plant relative density (RD) and abundance of gut microbiota described through curve fitting: *Nymphoides peltate* with Firmicutes (**a**), *UCG-005* (**b**), *Paenisporosarcina* (**c**) and *Christensenellaceae_R-7_group* (**d**). *Phragmites australis* with *Psychrobacillus* (**e**). *Setaria viridis* with Firmicutes (**f**) and *UCG-005* (**g**). *Rumex patientia* with Bacteroidota (**h**) and *UCG-005* (**i**). Each data point represents one sample. The red line represents the fitted curve. The yellow area is the 95% confidence band.

Spearman’s rank correlation analysis showed that the main foods of Père David’s deer had an extremely significant correlation with intestinal bacteria (Figure 4 and Figure 5). Among the main intestinal bacteria identified, Firmicutes (*p* = 0.005), *UCG-005* (*p* = 0.024), *Paenisporosarcina* (*p* = 0.003), *Christensenellaceae_R-7_group* (*p* = 0.021), and *unclassified_c__Clostridia* (*p* = 0.018) had an extremely significant correlation with the RD of *N. peltata* (Figure 5). The RD of some plants showed a negative correlation with the relative abundance of the main flora in the gut, including *P. australis* with Firmicutes (*p* = 0.029), Cyanobacteria (*p* = 0.008), and *Psychrobacillus* (*p* = 0.011); *S. viridis* with Firmicutes (*p* = 0.040), *UCG-005* (*p* = 0.046), and *Paenisporosarcina* (*p* = 0.027); and *M. spicatum* with *UCG-005* (*p* = 0.003), *norank_f__UCG-010* (*p* = 0.022), *norank_f__Eubacterium_coprostanoligenes_group* (*p* = 0.050), *Christensenellaceae_R-7_group* (*p* = 0.005), *unclassified_c__Clostridia* (*p* = 0.017), *Ruminococcus_torques_group* (*p* = 0.011), and *Alistipes* (*p* = 0.030). In addition, five plant species showed significant positive correlations with the relative abundance of *Psychrobacillus*, including *H. scandens* (*p* = 0.028), *S. nigrum* (*p* = 0.035), *C. album* (*p* = 0.022), *P. orientale* (*p* = 0.039), and *A. carvifolia* (*p* = 0.034). The relative abundance of *Psychrobacter* in the gut of Père David’s deer was positively correlated with the RD of *H. scandens* (*p* = 0.030) and *S. glauca* (*p* = 0.007).

## 4. Discussion

As Père David’s deer are being reintroduced to their original habitat, they face numerous challenges, including a change in dietary conditions, which exerts significant pressure on their survival. Consequently, they must continually adapt their foraging strategies to relieve the stress caused by environmental changes. Previous studies have demonstrated that changes in the composition of foods could play an important role in shaping the composition of the gut microbial community. A shift in an animal’s diet toward the natural plants of the reintroduction habitat along with a relatively stable pattern of the gut microbiome can serve as indicators for determining the appropriate time for translocation [13]. In this study, we focused on the fecal residues of Père David’s deer and analyzed the relationship between the composition of the gut microbiota and the diet, with the goal of providing a reference for the ex situ protection and release of animals from captivity to the wild historical habitat.

The results showed that the main food sources of Père David’s deer from Tianjin Qilihai Wetland in early winter were *N. peltata*, *P. australis*, *S. viridis*, and *T. orientalis*. *N. peltata*, characterized by strong resistance to cold temperatures from the mucilages secreted by the roots [32], which is the dominant phytoplankton species in Tianjin Qilihai Wetland during the harsh winter. This plant has a high proportion of nitrogen (330 μmol/m^2^) and phosphorus (56 μmol/m^2^) elements [33], and this study provides the first evidence that this rich, nutritious plant is preferred by Père David’s deer. Optimal foraging theory states that animals preferentially choose higher-energy food, which can explain why Père David’s deer frequently consume *N. peltata* owing to its high level of crude protein. In addition, palatability is another important factor determining dietary preference. Overall, our results suggest that *N. peltata* represents an important winter food for Père David’s deer.

*P. australis* was identified as another main food ingested by Père David’s deer, and its content was significantly negatively correlated with the most abundant bacterial genus identified in the fecal samples, *Psychrobacillus* (*p* = 0.011). *P. australis* has a low content of crude protein but is rich in acid and neutral detergent fibers [34]. When the content of crude protein reaches 16%, the food is considered to meet the basic requirements of Père David’s deer [35]. A high content of neutral and acidic detergent fibers reduces the digestibility of the forage by herbivores [36]. Therefore, we speculate that *P. australis* may be more readily consumed by Père David’s deer when plant resources are scarce in winter. Despite its low nutritional value, Père David’s deer would still consume a greater amount of this plant, in line with the foraging strategy of minimizing foraging time [37]. In addition, previous micro-histological analyses of cuticular structure fragments of common reed roots identified in fecal samples suggested that Père David’s deer selectively consume different parts of the plant during different seasons depending on plant growth [38,39,40] (Table 2).

*S. viridis* was identified as the third most highly consumed food; however, a previous study showed that this plant is not of the type Père David’s deer prefer to eat, and is only consumed when resources are particularly scarce during winter [9]. Similarly, we found that the RD of *S. viridis* was negatively correlated with the relative abundance of Firmicutes, the most dominant phylum in the gut microbiome, and with the important genus *UCG-005* of this phylum. This may be attributed to the fact that *S. viridis* contains relatively high acidic and neutral detergent fibers (Table 2), which are widely recognized as indicators of digestibility and feeding potential for ruminants [39].

*T. orientalis,* with a high crude protein and low insoluble polysaccharide content (Table 2), was also identified as a beneficial food source for Père David’s deer to obtain sufficient energy to survive in early winter. The RD of *T. orientalis* accounted for more than 10% of the total food composition, and we speculated that nutritional value factors such as crude protein, crude fat, and polysaccharides would be important factors influencing its intake by Père David’s deer in winter, in line with the previous findings by Wang et al. [21].

In this study, the dominant phyla in the gut microbiota of Père David’s deer were identified as Firmicutes and Bacteroides, which is consistent with findings for other ruminants and Père David’s deer living in other areas of China, such as Jiangsu [19,29,41], and Beijing and Hubei [42]. The same proportion of these bacteria was reported in the gut microbiome of the forest musk (*Moschus berezovskii*), captive alpine musk (*Moschus sifanicus*) [43], and wild blue sheep (*Pseudois nayaur*) [44]. Ruminants are herbivores and are thus highly dependent on the microbial community to absorb the nutrients from plants [44]. The genera of Firmicutes encode various enzymes related to energy metabolism that metabolize a variety of substances [45,46], and many of these microorganisms also have the ability to degrade carbohydrates such as cellulose and starch as well as fats [43]. Moreover, many microorganisms in the phylum Bacteroidota play roles in the degradation of macromolecular compounds such as proteins and carbohydrates [47]. Therefore, the high relative abundance of Firmicutes and Bacteroidota in the intestine of Père David’s deer may be related to its main plant source during the wintering period.

Firmicutes were found to be the dominant phylum with the highest abundance in the gut microbiota of Père David’s deer, which was similar to the results of previous studies [19,29,41]. The relative abundances of Firmicutes (*p* = 0.005) and *UCG-005* (*p* = 0.024) were significantly positively correlated with the RD of *N. peltata*. *UCG-005*, a genus in the phylum Firmicutes, could help Père David’s deer digest the high contents of cellulose, protein, and fat in *N. peltata* [32,33,48,49]. *Norank_f__UCG-010*, another member of Firmicutes, has been reported to be an important energy source for ruminants [50,51]. *Norank_f_Eubacterium_coprostanoligenes_group* contributes to the absorption and utilization of fat [52]. This study showed that the relative abundance of *Christensenellaceae_R-7_group* was positively correlated with the RD of *N. peltata*, which is considered to be an important genus for glucose and protein metabolism in ruminants [53]. *Monoglobus* was reported to be closely related to pectin degradation in the plant cell wall structure [54]. In summary, specific genera may facilitate the digestion and absorption of different nutrients by Père David’s deer. The dominant genera identified in this study, such as *UCG-005*, *Christensenellaceae_R-7_group*, and *norank_f_Eubacterium_coprostanoligenes_group*, were consistent with those reported by Sun et al. for free-ranging populations and by Wang et al. for captive populations [19,41]. However, the RD of *R. patientia* was negatively correlated with many of the dominant genera in Firmicutes, suggesting that this food may not be an ideal target for foraging.

The present study showed that the gut microbiota of Père David’s deer had a higher ratio of Firmicutes/Bacteroidota (F/B) in early winter compared to that previously calculated in the summer and autumn for a wild population in Jiangsu [19,41] (Table S3). A higher F/B is typically associated with better absorption of nutrients, which can improve the utilization of proteins and lipids [55]. Accordingly, we presumed that the gut microbiota composition enables individuals to increase the efficiency of energy intake to adapt to the change in plant resources in winter [56]. This is similar to the results of previous studies on forest musk and alpine musk [43,56].

*Psychrobacillus* from Firmicutes was the most abundant genus identified in the fecal samples, which was consistent with the study of You et al. on white-lipped deer. *Psychrobacillus* was also found to be significantly enriched during the withering season compared to the grassy season [57], and it was not identified in the gut microbiota of captive white-lipped deer [58]. Another genus of Firmicutes, *Paenisporosarcina*, has been identified in extremely cold regions and is considered to be an important plant rhizobium bacterium with frost resistance [59]. Our analysis showed that the abundance of this genus was positively correlated with the RD of *N. peltata*, containing mucilage with an antifreeze effect [32]. Based on this association, we hypothesize that the enrichment of *Paenisporosarcina* in the gut microbiota may be derived from the natural plants consumed by Père David’s deer in winter.

The increase in the relative abundance of Proteobacteria and Actinobacteriota indicates the health status of Père David’s deer under a condition of food reduction in early winter. *Pseudomonas* and *Acinetobacter*, which belong to Proteobacteria, are regarded as indicators of dysbiosis [60]. As these are conditionally pathogenic bacteria that can cause an inflammatory response in the host organism [57], these two genera may increase the potential for a pathological effect in Père David’s deer. The diminished plant resources in the field during winter [19], resulted in an unstable diet structure among Père David’s deer individuals, which may lead to significant alterations of intestinal bacteria in the wild.

## 5. Conclusions

This study represents the first investigation into the relationship between dietary composition and gut microbiota characteristics of ex situ conserved Père David’s deer in Tianjin. Microscopic observation revealed that *N. peltata*, which is prevalent in the Tianjin Qilihai Wetland during winter, was the dominant component identified from fecal samples. By high-throughput sequencing and correlation analysis, the composition and diversity of the gut microbiota were found to be closely associated with food selection, indicating that nutrients derived from edible plants could significantly influence the relative abundance of gut bacteria at the phylum/genus levels. The diverse gut microbiota within the ex situ conservation population, along with its dietary associations, demonstrate the robust potential for survival and adaptability, thereby enhancing prospects for successful population restoration and complete rewilding efforts. However, the sample size of this study was quite small, which was not sufficient to provide direct evidence of the influence of changes in food on the gut bacterial community. Future research should aim to elucidate the relationship between food consumption and gut bacteria to provide essential data for the full reintroduction of this endangered species and recovery of wild populations, ultimately contributing to biodiversity maintenance and ecological conservation.

## Figures and Tables

**Table 1:** Relative density (RD) of various identified plant species in the overall sample.

Rank	Species	Family	RD (%)	Type
1	*Nymphoides peltata*	Gentianaceae	35.14 ± 13.30	Main
2	*Phragmites australis*	Poaceae	26.62 ± 12.84	Main
3	*Setaria viridis*	Poaceae	12.11 ± 5.03	Main
4	*Typha orientalis*	Typhaceae	10.85 ± 4.26	Main
5	*Rumex patientia*	Polygonaceae	2.55 ± 2.64	Common
6	*Myriophyllum spicatum*	Haloragidaceae	2.38 ± 2.88	Common
7	*Humulus scandens*	Moraceae	2.38 ± 2.09	Common
8	*Suaeda glauca*	Chenopodiaceae	2.16 ± 2.90	Common
9	*Bolboschoenus yagara*	Cyperaceae	1.95 ± 3.34	Common
10	*Solanum nigrum*	Solanaceae	1.71 ± 2.08	Common
11	*Kochia scoparia*	Chenopodiaceae	0.77 ± 1.50	Occasional
12	*Echinochloa crus-galli*	Poaceae	0.34 ± 0.56	Occasional
13	*Chenopodium album*	Chenopodiaceae	0.28 ± 0.76	Occasional
14	*Polygonum orientale*	Polygonaceae	0.19 ± 0.64	Occasional
15	*Digitaria sanguinalis*	Poaceae	0.17 ± 0.55	Occasional
16	*Metaplexis japonica*	Asclepiadaceae	0.16 ± 0.60	Occasional
17	*Polygonum aviculare*	Polygonaceae	0.11 ± 0.33	Occasional
18	*Artemisia carvifolia*	Asteraceae	0.09 ± 0.23	Occasional
19	*Lysimachia barystachys*	Poaceae	0.06 ± 0.15	Occasional

**Table 2:** Chemical composition of edible plants in Père David’s deer diet (% dry matter).

Species	*P. australis*	*S. viridis*	*T. orientalis*
Crude protein	2.84	11.89	10.8
Crude oil	1.44	1.49	2.9
Neutral detergent fiber (NDF)	77.78	65.58	-
Acid detergent fiber (ADF)	45.9	40.91	-
Acid detergent lignin (ADL)	-	4.61	17.1
Cellulose content (ADF—ADL)	-	-	35.6
Hemicellulose content (NDF—ADF)	-	-	19.9
Reference	[38]	[39]	[40]

## Data Availability

The datasets for this study can be found in the NCBI Sequence Read Archive (SRA) under accession number PRJNA1007256.

## Supplementary Materials

The following supporting information can be downloaded at: https://www.mdpi.com/article/10.3390/ani14050728/s1, Table S1: Basic information of sampled plants; Table S2: Sample sequencing information statistics; Table S3: Comparison of Firmicutes/Bacteroidota between studies.
