# Supplementary material for: Impact of Environmental Food Intake on the Gut Microbiota of Endangered Père David’s Deer: Primary Evidence for Population Reintroduction
Source: Animals (Basel). 2024 Feb 27;14(5):728. doi: 10.3390/ani14050728 (PMC10931168; doi:10.3390/ani14050728)
Supplement: Supplementary file 1 [file animals-14-00728-s001.zip › animals-2730716-supplementary.pdf]

Supplementary Table S1. Basic information of sampled plants

No.	Sampling Time	Species	Family	Sampling Part
1	2022.09	*Najas marina*	Najadaceae	Roots, stems, leaves
2	2022.07	*Acalypha australis*	Poaceae	Stems, leaves
3	2022.07	*Carex scabrifolia*	Poaceae	Stems, leaves
4	2022.07	*Setaria viridis*	Poaceae	Stems, leaves, spikes
5	2022.07	*Lysimachia barystachys*	Poaceae	Stems, leaves, spikes
6	2022.07	*Phragmites australis*	Poaceae	Roots, stems, leaves, spikes
7	2022.07	*Digitaria sanguinalis*	Poaceae	Stems, leaves, spikes
8	2022.09	*Leymus chinensis*	Poaceae	Dry grass
9	2022.07	*Echinochloa crusgalli*	Poaceae	Stems, leaves, spikes
10	2022.07	*Abutilon theophrasti*	Malvaceae	Stems, leaves
11	2022.07	*Helianthus tuberosus*	Asteraceae	Stems, leaves, flowers
12	2022.07	*Ixeris polycephala*	Asteraceae	Stems, leaves, flowers
13	2022.07	*Artemisia carvifolia*	Asteraceae	Stems, leaves
14	2022.07	*Cirsium setosum*	Asteraceae	Stems, leaves, flowers
15	2022.07	*Kochia scoparia*	Chenopodiaceae	Stems, leaves
16	2022.07	*Suaeda glauca*	Chenopodiaceae	Stems, leaves
17	2022.07	*Chenopodium album*	Chenopodiaceae	Stems, leaves
18	2022.07	*Rumex patientia*	Polygonaceae	Stems, leaves
19	2022.07	*Polygonum aviculare*	Polygonaceae	Stems, leaves
20	2022.11	*Polygonum orientale*	Polygonaceae	Dry grass
21	2022.11	*Polygonum lapathifolium*	Polygonaceae	Dry grass
22	2022.09	*Nymphoides peltata*	Gentianaceae	Roots, stems, leaves, flowers
23	2022.07	*Cynanchum chinense*	Asclepiadaceae	Stems, leaves, flowers
24	2022.07	*Metaplexis japonica*	Asclepiadaceae	Stems, leaves
25	2022.07	*Portulaca oleracea*	Portulacaceae	Stems, leaves
26	2022.11	*Solanum nigrum*	Solanaceae	Dry grass
27	2022.07	*Humulus scandens*	Moraceae	Stems, leaves
28	2022.07	*Bolboschoenus yagara*	Cyperaceae	Stems, leaves, spikes
29	2022.07	*Typha orientalis*	Typhaceae	Stems, leaves
30	2022.09	*Myriophyllum spicatum*	Haloragidaceae	Roots, stems, leaves

Supplementary Table S2. Sample sequencing information statistics

Sample\Info	Seq_num	Base_num	Mean_length	Min_length	Max_length
SF01	270283	113827149	421.140616	302	448
SF02	230382	97706922	424.108316	204	434
SF03	245811	103988796	423.043704	235	452
SF04	281643	117965135	418.846323	277	452
SF05	259346	109095343	420.655584	291	450
SF06	263236	110803611	420.92879	249	431
SF07	272292	112446104	412.961468	258	449
SF08	276473	114079347	412.623826	326	451
SF09	249059	104674297	420.279119	337	446
SF10	255500	108609095	425.084521	304	432
SF11	232233	97703170	420.711828	219	452
SF12	271172	111664851	411.786066	232	431
SF13	253238	106398700	420.152979	328	435
SF14	221532	94533750	426.72729	328	449
SF15	248546	103965404	418.294416	229	431

Supplementary Table S3. Comparison of Firmicutes/Bacteroidota between fecal samples of wild Père David's deer population from different studies

Collection site	Number	Time	Firmicutes (%)	Bacteroidota (%)	F/B	Reference
Tianjin	15	Early winter	68.0	17.7	3.8	Our work
Jiangsu	95	Summer	46.6	38.3	1.2	[19]
	113	Winter	51.9	41.5	1.3	
	6	Autumn	43.2	19.0	2.3	[41]
